# Commentary on: A Case Report of Capsular Contracture Immediately Following COVID-19 Vaccination

**DOI:** 10.1093/asjof/ojab026

**Published:** 2021-06-21

**Authors:** Thomas C Wiener

Please watch a video commentary on “A Case Report of Capsular Contracture Immediately Following COVID-19 Vaccination,” ^1^ featuring Thomas C. Wiener, MD (Video).

## Supplementary Material

ojab026_Supplementary_Data
